# Supplementary material for: Simultaneous bicolor interrogation in thulium optical clock providing very low systematic frequency shifts
Source: Nat Commun. 2021 Aug 27;12:5171. doi: 10.1038/s41467-021-25396-8 (PMC8397736; doi:10.1038/s41467-021-25396-8)
Supplement: Supplementary file 1 — Supplementary Information [file 41467_2021_25396_MOESM1_ESM.pdf]

Supplementary Material to Simultaneous bicolor interrogation in thulium optical clock providing very low systematic frequency shifts

A. Golovizin[1*], D. Tregubov[1], E. Fedorova[1], D. Mishin[1],
D.Provorchenko[1], K. Khabarova[1,2], V. Sorokin[1], N. Kolachevsky[1,2]

[1] *P.N. Lebedev Physical Institute,*
Leninsky prospekt 53, 119991 Moscow, Russia

[2] *Russian Quantum Center,*
Bolshoy Bulvar 30, bld. 1,
Skolkovo IC, 121205 Moscow, Russia

[*] *email: artem.golovizin@gmail.com*

(Dated: August 2, 2021)

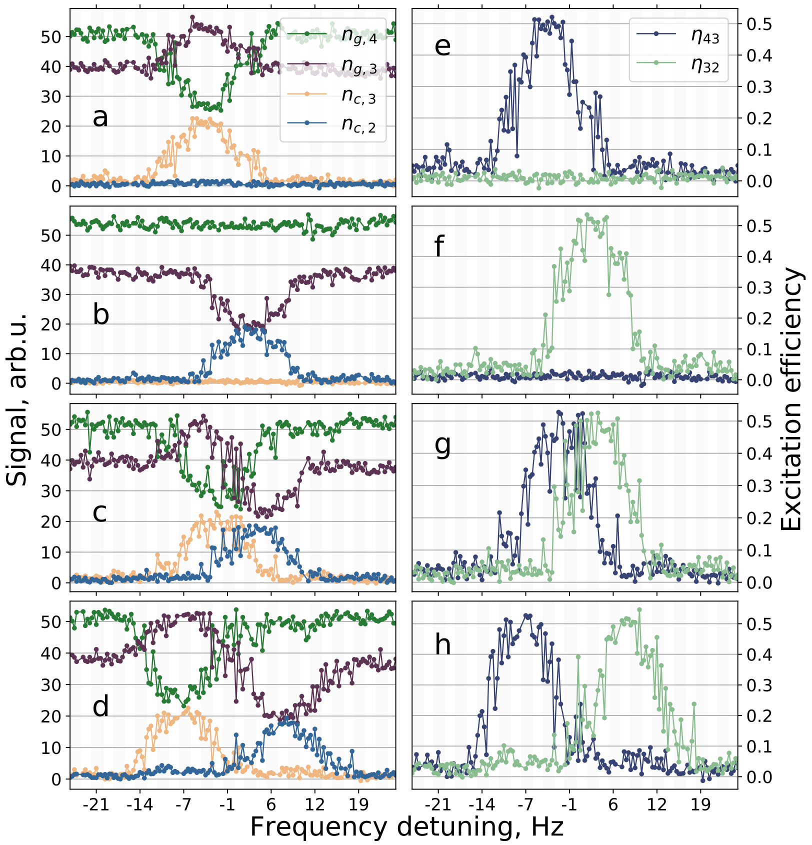

Supplementary Figure 1. Simultaneous measurement of excitation probabilities. Plots a–d show the raw data, plots e–f show excitation probabilities deduced from the corresponding raw data using Equations (15) and (16) from the Methods section. The first row (a and e) represents the case when the 3-2 excitation field is switched off and only the 4-3 transition is excited. The second row (b and f) shows the case when only the 3-2 transition is excited. In the third (c and g) and fourth (d and h) rows, both transitions are scanned using the simultaneous excitation procedure. The deliberately introduced additional offset between the two interrogating laser fields (3 Hz in the 3rd row and 9 Hz in the 4th row) defines the time sequence of passing through the two resonance curves. One can see that the resonance profiles are readily recovered in both the case of overlapped resonances and the case of separate resonances. Color schemes are identified in the top plots of each column.

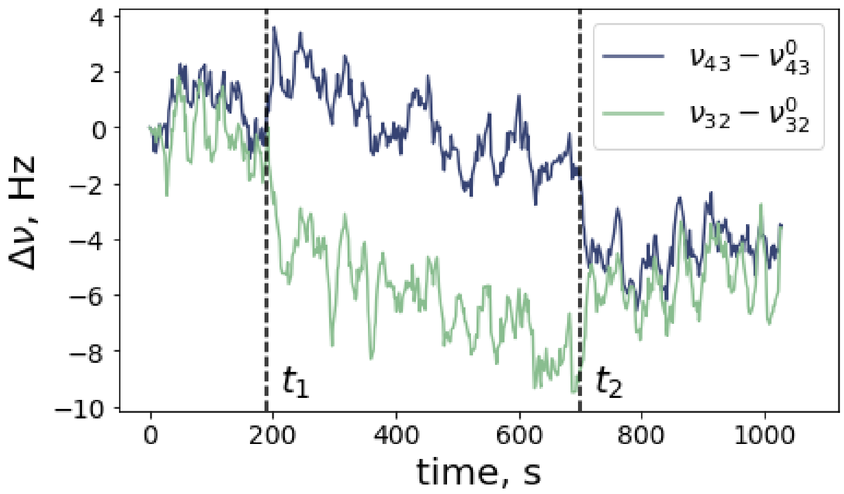

Supplementary Figure 2. Response of two parallel frequency lock channels ν_{43} and ν_{32} to an instantaneous change of the bias field B_0 from 218 mG to 231 mG at $t_1 = 190$ s and back at $t_2 = 700$ s. ν_{43}^0 and ν_{32}^0 are the corresponding AOM frequencies at $t = 0$. Laser frequency fluctuations and drift are common for both data sets.

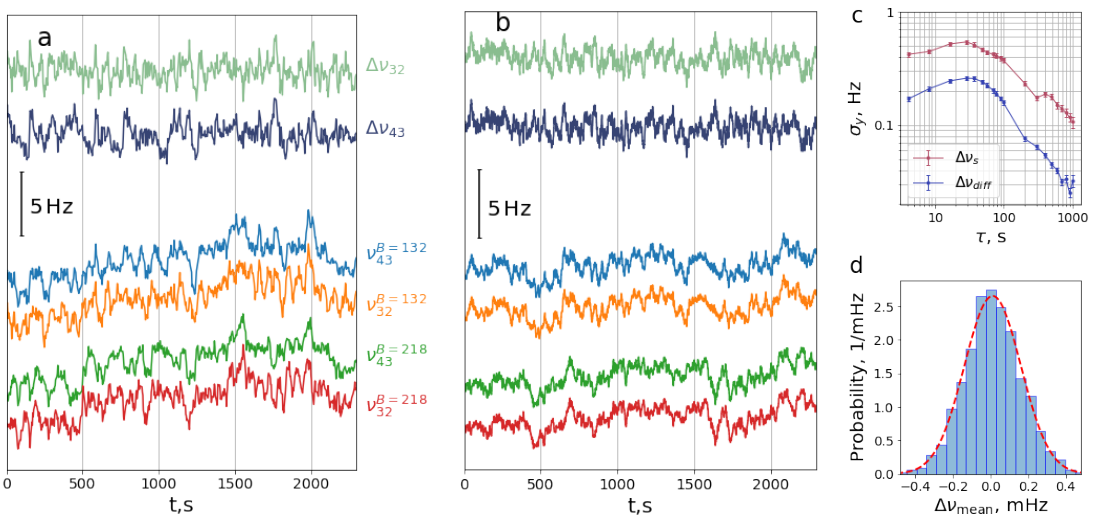

Supplementary Figure 3. Experimental frequency traces for $\Delta\nu_{43}$ and $\Delta\nu_{32}$ and individual traces $\nu_{43}(B^{\mathrm{m}})$, $\nu_{32}(B^{\mathrm{m}})$, $\nu_{43}(B^{\mathrm{r}})$ and $\nu_{32}(B^{\mathrm{r}})$ for the bias magnetic fields $B_0^{\mathrm{r}} = 218\,\mathrm{mG}$ and $B_0^{\mathrm{m}} = 132\,\mathrm{mG}$ (a), and results of simulations (b). Each trace is shifted for visual representation. c) Comparison of the Allan deviation plots (experiment) for the synthetic frequency $\Delta\nu_{\mathrm{s}} = (\Delta\nu_{43} + \Delta\nu_{32})/2$ (red) and the differential frequency $\Delta\nu_{\mathrm{diff}} = (\Delta\nu_{43} - \Delta\nu_{32})/2$ (blue). Errorbars correspond to 1 s.d. statistical uncertainty. d) Histogram of the difference between the 3-2 clock transition line centers from digital frequency locks with the 4-3 interrogation field ON and OFF (simulation). A total of 3000 samples, each corresponding to 1 hour of data collection, were used for the analysis. According to a Gaussian fit (red dashed line), the mean difference equals $12\,\mu$Hz with a standard deviation of $150\,\mu$Hz.

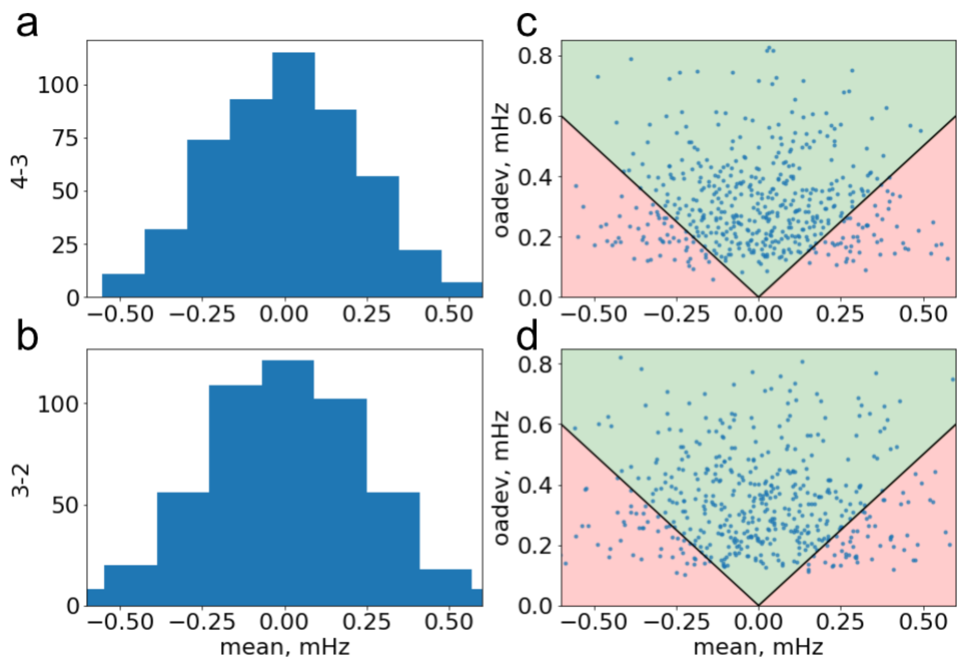

Supplementary Figure 4. Histograms of the frequency shifts of the digital locks for 4-3 (a) and 3-2 (b) transitions for simultaneous interrogation scheme. (c) and (d) show scatter plots of frequency shift and Allan deviation for every simulation run. The regions where the frequency shift is smaller (larger) than the Allan deviation are shown with green (red).

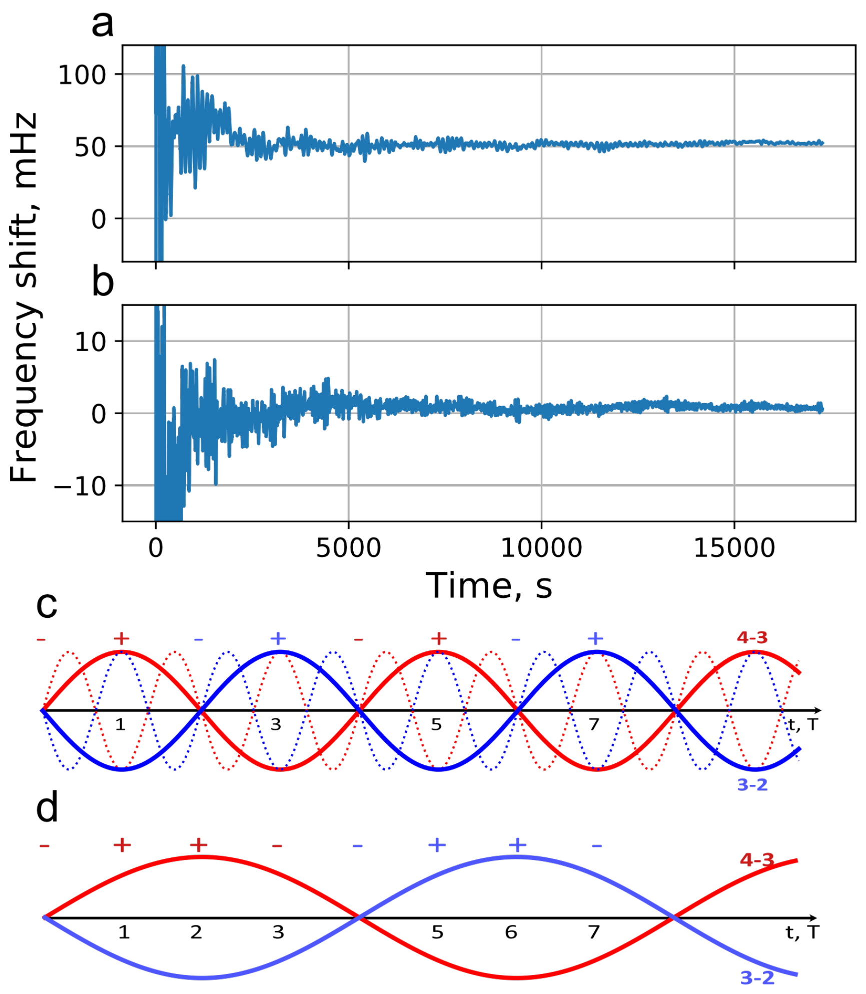

Supplementary Figure 5. Cumulative frequency shift of the synthetic frequency for sequential (a) and simultaneous (b) interrogation schemes under small oscillations of the bias magnetic field. (c) and (d) show influence of the bias magnetic field oscillations on the 4-3 (red) and 3-2 (blue) clock transition frequencies (amplitude is $\Delta\nu = 0.1\,\mathrm{Hz}$) for different probe schemes. "+" and "-" signs depict moments of interrogation of right and left slopes, correspondingly, with the color indicating which transition is probed in sequential scheme.

Parameter	Tm	Sr
$(1/h)\partial\tilde{\alpha}^{E1}/\partial\nu$	$1.6(4)\times10^{-14}$	$1.735(13)\times10^{-11}$
$\tilde{\alpha}^{\mathrm{qm}}/h$	$1.2\,\mathrm{mHz}$	$-0.962(40)\,\mathrm{mHz}$
$\tilde{\beta}/h$	$-1.5\,\mathrm{nHz}$	$-0.461(14)\,\mu\mathrm{Hz}$

Supplementary Table I. Comparison of lattice shift-related coefficients for Tm and Sr (from Ref. [1]).

REFERENCES

[1] Ushijima, I, Takamoto, M & Katori, H Operational magic intensity for Sr optical lattice clocks. *Phys. Rev. Lett.* **121**, 263202 (2018).
