# Supplementary material for: Simultaneous bicolor interrogation in thulium optical clock providing very low systematic frequency shifts
Source: Nat Commun. 2021 Aug 27;12:5171. doi: 10.1038/s41467-021-25396-8 (PMC8397736; doi:10.1038/s41467-021-25396-8)
Supplement: Supplementary file 3 — Description of Additional Supplementary Files [file 41467_2021_25396_MOESM3_ESM.pdf]

Description of Additional Supplementary Files

Supplementary Data 1: Description

Raw data for the figures in the main text and Methods section.

Supplementary Data 2: Description

Raw data for the figures in the Supplementary Information file.
